# Candidate genes revealed by a genome scan for mosquito resistance to a bacterial insecticide: sequence and gene expression variations

**DOI:** 10.1186/1471-2164-10-551

**Published:** 2009-11-21

**Authors:** Aurélie Bonin, Margot Paris, Guillaume Tetreau, Jean-Philippe David, Laurence Després

**Affiliations:** 1 Laboratoire d'Ecologie Alpine, CNRS-UMR 5553, Université Joseph Fourier, BP 53, 38041 Grenoble cedex 09, France

## Abstract

**Background:**

Genome scans are becoming an increasingly popular approach to study the genetic basis of adaptation and speciation, but on their own, they are often helpless at identifying the specific gene(s) or mutation(s) targeted by selection. This shortcoming is hopefully bound to disappear in the near future, thanks to the wealth of new genomic resources that are currently being developed for many species. In this article, we provide a foretaste of this exciting new era by conducting a genome scan in the mosquito *Aedes aegypti *with the aim to look for candidate genes involved in resistance to *Bacillus thuringiensis *subsp. *israelensis *(*Bti*) insecticidal toxins.

**Results:**

The genome of a *Bti*-resistant and a *Bti*-susceptible strains was surveyed using about 500 MITE-based molecular markers, and the loci showing the highest inter-strain genetic differentiation were sequenced and mapped on the *Aedes aegypti *genome sequence. Several good candidate genes for *Bti*-resistance were identified in the vicinity of these highly differentiated markers. Two of them, coding for a cadherin and a leucine aminopeptidase, were further examined at the sequence and gene expression levels. In the resistant strain, the cadherin gene displayed patterns of nucleotide polymorphisms consistent with the action of positive selection (e.g. an excess of high compared to intermediate frequency mutations), as well as a significant under-expression compared to the susceptible strain.

**Conclusion:**

Both sequence and gene expression analyses agree to suggest a role for positive selection in the evolution of this cadherin gene in the resistant strain. However, it is unlikely that resistance to *Bti *is conferred by this gene alone, and further investigation will be needed to characterize other genes significantly associated with *Bti *resistance in *Ae. aegypti*. Beyond these results, this article illustrates how genome scans can build on the body of new genomic information (here, full genome sequence and MITE characterization) to finally hold their promises and help pinpoint candidate genes for adaptation and speciation.

## Background

In the past few years, evolutionary biologists have increasingly bet on population genomics approaches to study the genetic basis of adaptation and speciation. Genome scans have flourished in the literature, providing valuable insight into the genetics of local adaptation [1,2], sympatric speciation [3], host race or ecotype differentiation [4-7], and response to climate change or exotic invasions [8,9], among others. In most cases, however, population genomics alone fell short of pinpointing the specific gene(s) or mutation(s) targeted by selection during the adaptation or speciation process [10,11].

One of the main reasons for this recurring setback is the present lack of genomic resources for most examined species. For example, due to the absence of more powerful alternatives, many population genomics studies rely on genetic markers such as AFLPs (Amplified Fragments Length Polymorphisms) [11,12], which can generally be obtained easily for any organism. Unfortunately, such markers present the double disadvantage of being anonymous and of falling predominantly in non-coding regions of the genome, i.e. far from potential candidate regions for adaptation and speciation [10]. Moreover, when genomic sequences are scarce or poorly annotated, identifying candidate genes in the vicinity of markers showing a selection signature represents a daunting task that few researchers have undertaken so far (but see [13]). Yet, we predict that these limitations are bound to disappear in the near future, especially with the increasing use of next-generation sequencing technologies. In this article, we aim at providing a foretaste of this exciting new era by illustrating how new genomic tools can help unravel the genetic basis of mosquito resistance to *Bacillus thuringiensis *subsp. *israelensis *(*Bti*) insecticidal toxins.

Considered as a safe alternative to chemical insecticides, the bio-insecticide *Bti *is widely used worldwide for mosquito control [14]. *Bti *toxicity is mainly conferred by three Cry toxins (Cry4A, Cry4B and Cry11A) and one Cyt toxin (Cyt1A), which aggregate in a proteic crystal produced during sporulation of the bacteria [15]. *Bti *is usually sprayed in mosquito breeding sites as a mixture of spores and toxins, which is ingested together with organic detritus by developing larvae. In the larval midgut, *Bti *toxins are first activated by protease/trypsin-like enzymes or aminopeptidases [16]. Then, they bind to specific receptors of the midgut cells (Cry toxins), or directly interact with the cell membrane (Cyt1A toxin), ultimately causing pore formation and cell lysis [16]. Cy1A is also known to act synergistically with Cry toxins, increasing the overall toxicity of the *Bti *mixture [17,18]. Due to the complexity of *Bti *toxicity mechanisms, some have argued that resistance to *Bti *would likely require adaptive mutations in several genes [19,20]. This argument is reinforced by the fact that only a handful of studies have observed evidence of laboratory or natural resistance to *Bti *in mosquito [19,21-23]. Because of the probable multilocus nature of *Bti *resistance, population genomics appears to be an approach of choice to identify genes involved in this process.

Here, we describe the application of population genomics to the search for candidate genes for resistance to a toxic leaf litter containing *Bti *spores. With the mosquito *Aedes aegypti *as a model, we first conducted a genome scan relying on about 500 MITE (Miniature Inverted-repeat Transposable Element)-derived markers expected to occur frequently in gene-rich regions [24,25]. By combining the results of this genome scan with data from the publicly available genome sequence of *Ae. aegypti*, we were then able to localize two good candidate genes for *Bti *resistance. Finally, these two genes were further analyzed at the sequence and gene expression levels in order to determine if selection was indeed a driving force in their evolution.

## Results

### Genome scan and identification of outlier loci presumably influenced by selection

Our search for candidate genes linked to *Bti *resistance was conducted in two *Aedes aegypti *strains differing drastically in their susceptibility to *Bti *as well as to individual *Bti *toxins (see the Methods part for resistance ratios). The genome of *Ae. aegypti *was screened using a variant of the DArT (Diversity Arrays Technology) procedure, where motifs of a particular MITE (Miniature Inverted-repeat Transposable Element) family called *Pony *served as primer anchors for PCR amplification. In total, 476 biallelic dominant markers were surveyed for 29 individuals in each mosquito strain, revealing a particularly high genetic differentiation between strains (mean *Fst *= 0.556). This strong genetic structure was not surprising given the history of the two strains, and in particular the recent bottleneck experienced by the resistant strain (Additional file 1). However, high neutral *Fst *values are expected to reduce the power of methods revealing outlier loci potentially under selection on the basis of an atypically high genetic differentiation. For example, the application of the program Dfdist [26] to our data detected only one locus departing from neutral expectations for α = 1%, because the neutral envelope included almost the entire range of possible differentiation values (Additional file 2). As a result, we adopted a different strategy and retained as outliers those loci for which alternative phenotypes (fragment presence/absence) were fixed or nearly fixed in each strain. A total of 70 such markers were sequenced for further analyses.

### Outlier sequencing and localization in the genome

Among the outlier sequences obtained, one pair differed only by a gap and another one by only a mutation, resulting in a redundancy rate of 2.86%. After trimming the primer sequence and the *Pony *motif, the 68 unique marker sequences (GenBank accession no. FJ231034-FJ231090; sequences shorter than 50 bp could not be deposited) had an average size of 185.2 bp (range 16-868 bp). Of these unique sequences, 41 could be assigned to a unique position in the *Aedes aegypti *genome, and all but two of these positions were found on different supercontigs (Additional file 3). Six sequences were situated on the same supercontig as a candidate gene for *Bti *resistance (DArT_102, DArT_318, DArT_400, DArT_415, DArT_432 and DArT_467), and two of them (DArT_432 and DArT_467) co-localized with the same gene (a cadherin). It had to be noted that physical distances between candidate genes and outlier markers situated on the same supercontig were considerable, ranging from 97907 bp (cadherin and DArT_467) to more than 300 Mbp (glycosyltransferase and DArT_415). According to these results, the cadherin gene (CAD, VectorBase Gene ID AAEL001196) turned out to be a serious candidate for *Bti *resistance because two outlier markers pointed towards it, one of them at the shortest distance recorded in this study. This gene, which codes for a possible toxin-binding receptor [27], was thus selected for further investigation at the sequence and expression levels. We also focused on the leucine aminopeptidase gene (LAP, VectorBase Gene ID AAEL001649) because of its potential implication in *Bti *toxin activation [28].

### Candidate gene sequence analysis

**Figure 1:**
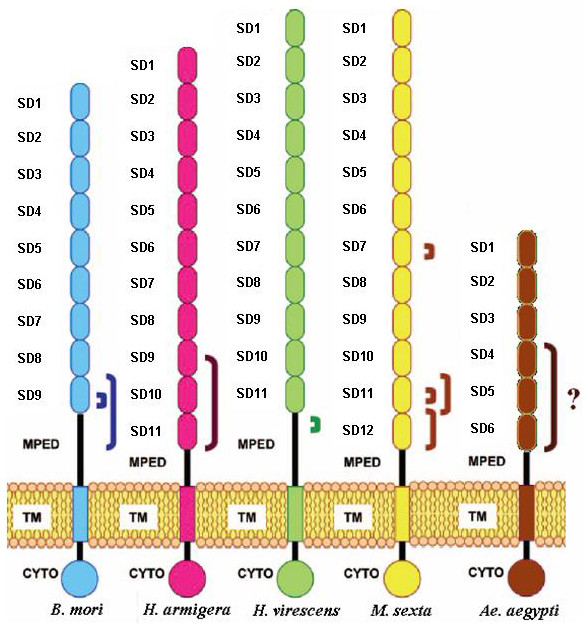
**Comparison between lepidopteran cadherin-like proteins and the *Aedes aegypti *cadherin studied here**. Cadherin-like proteins are constituted of different domains: SD, subdomain; MPED, membrane-proximal extracellular domain; TM, transmembrane domain; CYTO, cytoplasmic domain. Only features present in the mature form of the protein are outlined here. Known and putative Cry binding sites characterized in lepidopterans are indicated by parentheses. *B. mori*, *Bombyx mori*; *H. armigera*, *Helicoverpa armigera*; *H. virescens*, *Heliothis virescens*; *M. sexta*, *Manduca sexta*; *Ae. aegypti, Aedes aegypti*. Adapted from Figure 10 in [27].

A total of 1,467 bp and 1,657 bp were sequenced for the CAD and LAP genes, respectively. All sequences were deposited into GenBank, with accession numbers GU066340 to GU066385. As shown in Table 1, the two candidate genes shared similarities: both had a higher genetic diversity in the susceptible than in the resistant strain even if more individuals were sequenced in this latter. This observation was consistent with the fact that both strains are separated by 18 generations of selection, and that the resistant strain experienced a strong bottleneck at generation 10 (Additional file 1). Both genes also showed similar numbers of haplotypes in each strain (13 and 12 haplotypes in the susceptible strain for CAD and LAP, respectively; and 5 haplotypes in the resistant strain for both genes). On the other hand, the two candidate genes differed by their overall level of genetic diversity and inter-strain differentiation. For example, the *Fst *value was 0.186 only for CAD vs. 0.321 for LAP. Likewise, the nucleotide diversity *π *estimated for LAP in the susceptible strain was about four times lower than that estimated for CAD, this ratio reaching 17 when considering the resistant strain. However, it has to be noted that polymorphisms were not uniformly distributed along the CAD gene sequence, some regions showing globally more variation (e.g. subdomain SD4 and interdomain SD4-SD5 of the protein; Figure 1).

**Table 1:** Measures of genetic diversity and divergence obtained for the two candidate genes

Gene/Domain		Strain	Fragment size (bp)	*N*	*Hap*	*S*	*Si*	*NS*	*Hd*	*π*	*θ*_*W*_	*k*	*K*	*Fst*
Cadherin (CAD)	Whole sequence*	Susceptible Resistant	1467	20 24	13 5	67 65	0 1	3 3	0.932 0.435	0.023 0.017	0.013 0.012	32.363 25.033	35.254	0.186
	
	SD2	Susceptible Resistant	75	22 24	3 2	2 2	0 0	0 0	0.567 0.391	0.013 0.010	0.007 0.007	0.961 0.783	1.136	0.233
	
	SD2-SD3	Susceptible Resistant	69	22 24	3 2	2 1	1 0	0 0	0.558 0.391	0.009 0.006	0.008 0.004	0.610 0.391	0.568	0.119
	
	SD3	Susceptible Resistant	268	22 24	3 2	12 8	4 0	1 0	0.541 0.391	0.016 0.012	0.012 0.008	4.264 3.130	4.705	0.214
	
	SD3-SD4	Susceptible Resistant	77	22 24	2 2	3 3	0 0	2 1	0.485 0.391	0.019 0.015	0.011 0.010	1.455 1.174	1.705	0.229
	
	SD4	Susceptible Resistant	279	22 24	4 2	18 14	4 0	0 0	0.571 0.391	0.026 0.020	0.018 0.013	7.273 5.478	8.080	0.211
	
	SD4-SD5	Susceptible Resistant	69	22 24	3 2	6 5	1 0	0 0	0.541 0.391	0.038 0.028	0.024 0.019	2.602 1.957	2.795	0.185
	
	SD5	Susceptible Resistant	246	10 22	4 4	17 14	1 0	1 1	0.733 0.398	0.034 0.020	0.024 0.016	8.422 5.039	8.164	0.176
	
	SD5-SD6	Susceptible Resistant	63	10 22	2 2	1 1	0 0	0 0	0.533 0.368	0.008 0.006	0.006 0.004	0.533 0.368	0.555	0.187
	
	SD6	Susceptible Resistant	279	10 22	2 2	15 15	0 0	1 1	0.533 0.368	0.029 0.020	0.019 0.015	8.000 5.519	8.318	0.187
	
	SUP6	Susceptible Resistant	42	10 22	2 2	1 1	0 0	0 0	0.533 0.368	0.013 0.009	0.008 0.007	0.533 0.368	0.555	0.187

Leucine aminopeptidase (LAP)		Susceptible Resistant	1657	22 24	12 5	29 6	0 0	9 2	0.900 0.688	0.006 0.001	0.005 0.001	10.069 2.025	8.909	0.321

*N*, number of alleles sampled; *Hap*, number of different haplotypes; *S*, number of segregating sites; *Si*, number of singletons; *NS*, number of non-synonymous mutations; *Hd*, haplotype diversity; *π*, nucleotide diversity (per site); *θ*_*W*_, Watterson's mutation parameter θ estimated from *S *(per site); *k*; average number of nucleotide differences within strain; *K*, average number of nucleotide differences between strains; *Fst*, genetic differentiation between strains.

* Some measures (e.g. *S *and *NS*) are different between the whole sequence and the total of all sequences because of a different number of sampled haplotypes.

These patterns of genetic diversity translated differently at the codon level. We found three non-synonymous sites in the CAD sequence, of which none was diagnostic of one strain in particular, whereas for the LAP sequence, only two of nine non-synonymous mutations were present in the resistant strain. Six non-synonymous sites, among which five could only be found in the susceptible strain, were noticeably situated in 5' and 3' untranslated regions and could thus affect transcript stability and/or translation.

Finally, the examination of haplotype repartition between strains allowed this picture to be completed. For the CAD gene, one haplotype present in all 12 sampled resistant individuals (18 haplotypes out of 24) existed only in 2 susceptible individuals (2 haplotypes out of 20). For the LAP gene, the most frequent haplotype in the resistant strain (11 haplotypes out of 24) was absent from the susceptible strain.

### Neutrality tests

For both candidate genes, we tested for deviation from neutral evolution in the resistant strain by applying different neutrality tests to the observed polymorphism data (Table 2). Tajima's *D *and Fay and Wu's *H *highlight a skew in the frequency distribution of variants, *H *giving more weight to high-frequency polymorphisms, whereas Fu and Li's D* and F* detect a discrepancy between either the total number of mutations (D*) or the average number of differences between two sequences (F*) and the number of singletons. As a result, each one of these tests is based on different diversity parameters and gives different information on the type of selection presumably in action. In this study, every test except Fay and Wu's *H *gave positive values for both genes (Table 2), which is consistent with the recent bottleneck experienced by the resistant strain. None of the tests was significant for LAP, whereas D*, F* and *H *remained significant for CAD even when accounting for the particular demographic history of the strain (Table 2). The positive values of D* and F* suggested a deficiency of recent (i.e. rare) mutations, whereas the negative *H *indicated an excess of high compared to intermediate frequency mutations.

**Table 2:** Results of the neutrality tests for the resistant strain

Neutrality test	Value	Significance according to coalescent simulations based on a large constant population size^a^	Significance according to coalescent simulations based on the known demographic history of the resistant strain^b^
**Cadherin (CAD)**			
Tajima's *D*	1.717	*p *< 0.01	n.s.
Fu and Li's D*	1.698	*p *< 0.001	*p *< 0.01
Fu and Li's F*	2.000	*p *< 0.001	*p *< 0.05
Fay and Wu's *H*	-50.370	*p *< 0.001	*p *< 0.01

**Leucine aminopeptidase (LAP)**			
Tajima's *D*	0.788	n.s.	n.s.
Fu and Li's D*	1.233	n.s.	n.s.
Fu and Li's F*	1.280	n.s.	n.s.
Fay and Wu's *H*	1.181	n.s.	n.s.

^a^, performed following the method of Hudson [70] implemented in DnaSP; ^b^, performed using the program ms [68].

### Gene expression analyses

**Figure 2:**
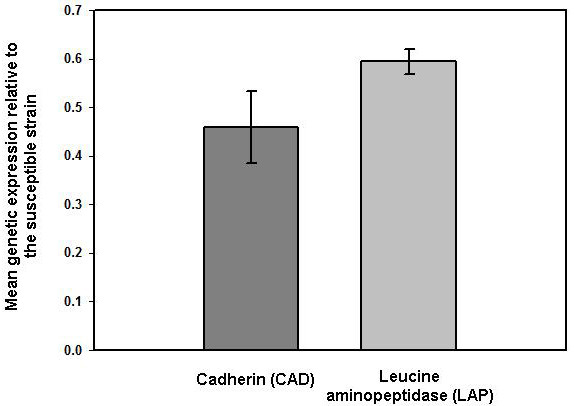
**Results of the expression analyses for the two candidate genes**. This figure illustrates, for each candidate gene, the mean gene expression in the resistant strain relative to that in the susceptible strain.

Real-time reverse-transcription PCR (RT-PCR) analyses revealed that levels of gene expression were reduced in the resistant strain compared to the susceptible strain for both candidate genes (2.17-fold and 1.68-fold under-expression for the CAD and the LAP genes, respectively; Figure 2). Non-parametric Mann-Whitney tests indicated that reduction in expression levels was significant for both genes (*p *= 0.017 and *p *= 0.002 for the CAD and LAP genes, respectively). However, only the expression fold change observed for the CAD gene (2.17) was higher than the two-fold change conservatively used as a significant threshold in expression studies [29].

## Discussion

### A MITE-based genome scan to search for candidate genes

The goal of this study was to identify genes conferring resistance to the bio-insecticide *Bacillus thuringiensis *subsp. *israelensis *(*Bti*) in an *Aedes aegypti *mosquito strain selected for several generations with a toxic leaf litter containing *Bti *spores. The genetic basis of *Bti *resistance is likely to be multigenic [19,20], and in *Ae. aegypti*, we listed as many as 160 serious candidates by virtue of their known function and/or proven association with *Bti *resistance in other species (e.g. [20,30,31]). As a result, we chose to tackle this study by adopting a population genomics approach. The underlying idea was to examine many MITE-derived DArT markers scattered in the genome to get an accurate estimate of the overall background (i.e. neutral) genetic differentiation between the selected (*Bti*-resistant) and control (*Bti*-susceptible) strains. This in turn allowed detecting markers with an atypically high inter-strain genetic differentiation, and thus possibly linked to a gene under positive selection due to *Bti *resistance. Our genome scan revealed an overall high level of neutral genetic differentiation between the two strains (mean *Fst *= 0.556), which is not unexpected in the light of the history of the resistant strain, and especially of the bottleneck it experienced at generation 10 (see Additional File 1). Yet, the *Fst *value obtained between the same two mosquito strains using AFLP markers is substantially lower (mean *Fst *= 0.114; Paris, pers. comm.). AFLP and DArT markers are biallelic, dominant and, to a certain extent, randomly distributed in the genome so they should provide similar genetic differentiation estimates. However, the DArT markers developed for the purpose of this study are intimately associated with a specific family of MITEs called *Pony*, whose characteristics might explain the discrepancy between the two *Fst *measures. *Pony *elements constitute about 1.1% of the genome of *Ae. aegypti *[25], and although their transposition mechanism is still unclear, it could be triggered by unfavorable environmental conditions as was shown for other MITEs in plants for example [24,32,33]. Several authors have even underlined the potential contribution of MITEs to rapid adaptations and, ultimately, genome evolution [24,34]. One can thus speculate that the environmental stress imposed by toxic leaf litter selection stimulated *Pony *transpositions, hence inflating estimates of inter-strain genetic differentiation as measured by our *Pony*-associated markers. Like many MITEs, *Pony *motifs are also known to frequently occur in the non-coding regions of genes [25]. Because of this last characteristic of *Pony *elements and of their high mutational potential possibly enhanced by stress, *Pony*-based DArTs are ideal random markers to explore the genome of *Ae. aegypti *and search for genes conferring resistance to *Bti*.

### DArT marker sequences and identification of candidate genes

We decided to sequence the MITE-based DArT markers showing the highest inter-strain differentiation (outliers), as those are the most likely to be linked to genes conferring resistance to *Bti*. Of the 70 sequences obtained, 41 (68.6%) matched to unique locations in the genome of *Ae. aegypti*, while the rest consisted of repeated sequences. As less than 40% of the genome of this species is constituted of single or low copy sequences [35], this result confirms the fact that MITE-based DArTs tend to fall near low-copy, possibly coding, sequences. The 41 genomic locations involved were all but two situated on different supercontigs of the *Ae. aegypti *full genome sequence, suggesting that there was no real bias in the distribution of our markers in the genome, at least for highly differentiated ones. Unfortunately, the *Ae. aegypti *genome has a weak gene density [35], and most of our outlier sequences (85.4%) fell in supercontigs with few or no putative genes. Furthermore, we recently performed a differential transcriptome analysis of *Ae. aegypti *larvae exposed to different xenobiotics. This study, based on the sequencing of millions of cDNA tags using next-generation sequencing, detected more than 2000 loci situated outside predicted genes and showing a significant transcription signal (David, pers. comm.). There is thus an obvious need for a better gene annotation in the *Ae. aegypti *genome, and one efficient strategy to tackle this task would be to concentrate the effort on regions of interest, like those showing signatures of selection.

The selection with toxic leaf litter was a recent event in the history of the resistant strain so linkage disequilibrium is probably extensive in the vicinity of selected genes [36]. In those conditions, detecting selection signatures in a genome is somewhat easier even with a relatively small marker density [37], but on the other hand, the existence of large haplotype blocks makes it more difficult to pinpoint the exact gene(s) under selection. In this study, the distance between outlier markers and candidate genes was considerable (between 100 and 3,000 Kb) and might exceed the window of linkage disequilibrium around selected genes. In other words, we might have overlooked closer genes genuinely responsible for the signatures of selection, but not already annotated or absent from our list of candidates.

### The cadherin and leucine aminopeptidase as candidate genes

Among the five candidate genes discovered in the proximity of outlier markers, we decided to examine further the cadherin (CAD) and leucine aminopeptidase (LAP) at the sequence and expression levels, to confirm or infirm the selection footprint. This step was all the more crucial since selection but also particular demographic histories can generate an atypically high genetic differentiation [38,39].

Neutrality tests are often used to verify the influence of selection in intraspecific sequence data [40,41]. However, they have often been criticized for their lack of power and their sensitivity to demographic events like bottlenecks or population expansions which can mimic selective effects [38,42]. In this study, this possible bias was overcome by performing tailor-made coalescence simulations based on the known demographic history of the resistant strain, in order to assess the significance of the tests. No firm evidence of selection was found at the nucleotide or expression level for the LAP gene, which rules out its implication in *Bti *resistance, at least as a gene with major effects. For the CAD gene, Fu and Li's D* and F* tests highlighted a deficit in rare mutations, which is usually the trademark of balancing selection [43]. Fay and Wu's *H *statistics indicated an excess of high compared to intermediate frequency mutations, which on the contrary suggests the spread of an advantageous mutation at a linked site (i.e. positive selection) [44]. Although those conflicting results might be hard to interpret at first glance, one has to keep in mind that the selection with the toxic leaf litter started only 20 generations ago. In addition, the resistant strain experienced a recent bottleneck which probably further eliminated low frequency variants. New mutations certainly have not had time yet to appear in the population, which would explain the positive D* and F* values. For the same reason, Tajima's *D *can be transiently positive right after a bottleneck [45]. As for the *H *statistics, it is not as sensitive to the loss of rare variants, so it is presumably more reliable here and we can reasonable think that the CAD gene shows genuine signs of positive selection. This evidence is further reinforced by the significant under-expression of this gene in the resistant strain. Cadherins are indeed known to bind to Cry toxins in other insect species [27] and to be involved in many cases of Cry resistance in insects (e.g. [46-48]). Here, under-expression of the cadherin gene could reduce the number of a certain type of Cry receptors and thus hinder pore forming, ultimately limiting susceptibility to *Bti*. Similarly, resistance to the toxin Cry1Ac has been shown to be linked to reduced levels of membrane receptors in the cotton pest *Heliothis virescens *[49], or in the cabbage moth *Plutella xylostella *[50]. Nevertheless, cadherins are usually specific to one particular Cry toxin [27], and it is unlikely that the CAD gene alone is responsible for resistance to *Bti *which is a mixture of several Cry toxins. One can thus hypothesize that this gene's effects supplement those of one or several other genes that remain to be identified.

## Conclusion

Beyond their implications for the understanding of the genetic mechanisms of *Bti *mosquito resistance, these results illustrate how genome scans can build on the body of new genomic information (here, full genome sequence and MITE characterization) to finally hold their promises and help pinpoint candidate genes for adaptation and speciation [10]. In the near future, a wealth of genomics tools will be available for a much wider range of species, mostly thanks to the rapid development of next-generation sequencing technologies [51]. We predict this new knowledge will boost in many respects the use of population genomics for the study of the genetic bases of adaptation and speciation. Several limitations of current genome scans will certainly be soon overcome, with for example (1) the development of new genetic markers allowing screening the genome more finely (e.g. [52]) or specifically targeting coding regions (e.g. [53]); (2) an easier access to outlier sequences as well as full genomic sequences serving as references to locate outlier loci and identify nearby candidate genes; and (3) a better gene annotation. In short, population genomics will at last have the means to meet our expectations when it comes to identify genes under natural or artificial selection.

## Methods

### Biological material

Two *Aedes aegypti *laboratory strains were compared for the purpose of this study: the Bora Bora reference strain, known to be susceptible to most insecticides, and a strain artificially selected for resistance to a decomposed tree leaf litter showing a high toxicity for mosquito larvae. This leaf litter had been collected in a mosquito pond in Eastern France three months after treatment for mosquito control, and has been proved to contain *Bacillus thuringiensis *subsp. *israelensis *(*Bti*) spores from commercial origin [54]. The use of this *Bti*-contaminated leaf litter in the selection experiments allowed mimicking the evolution of resistance to *Bti *in a situation close to field conditions. The susceptible and resistant strains were separated by 18 generations of selection (and by 20 generations in total), with a strong bottleneck at generation 10 (see Additional File 1 for more details on the demographic history of the resistant strain). The selection experiment and the bioassays implemented to monitor the evolution of resistance are described in [55]. At each generation, the lethal dose for 50% of the individuals after a 24 h-exposure (24 h-LD50) was determined for each strain using the Probit software [56]. After 18 generations of selection, the resistance ratios RR of the resistant strain (i.e., the ratio between the 24 h-LD50 values for the resistant and the susceptible strains, respectively) were 3.4-fold, 30.2-fold, 13.7-fold, 6.3-fold and 3-fold for the toxic leaf litter, Cry4A, Cry4B, Cry11A and Cyt1A toxins, respectively.

### DNA extraction and genome scan

The genomic DNA used for all subsequent molecular work was extracted from fresh fourth-instar mosquito larvae using the Qiagen DNeasy Tissue Kit and protocol (Qiagen). Prior to extraction, the larvae midgut was removed carefully to avoid bacterial contamination.

The classical protocol of the Diversity Array Technology (DArT) [57] was slightly modified so as to provide hundreds of good-quality markers scattered in the genome of *Ae. aegypti *and possibly associated to gene-rich regions [55]. Briefly, in a first step, genomic DNA was digested with restriction enzyme Bsp1286I and a specific adaptor was ligated to compatible ends. Restriction fragments including a particular *Ae. aegypti *MITE called *Pony *were PCR-amplified using a primer annealing to the adaptor sequence and a primer complementary to a conserved motif of the *Pony *element. PCR products obtained for all individuals of the two strains were pooled together and cloned to construct a DArT library containing a total of 6144 MITE-based clones. In a second step, a labelled target produced for each *Ae. aegypti *individual as described in the first step was hybridized to the library fragments spotted on a glass slide in order to reveal the polymorphic ones. Details of the protocol, in particular the adaptor and primer sequences used and the reproducibility rates, can be found in [55].

### Identification and sequencing of outlier loci potentially under selection

For each DArT marker obtained, allelic frequencies were estimated with the Bayesian method with non-uniform prior distribution [58] implemented in AFLP-SURV 1.0 [59]. Among those markers, we tracked those for which alternative phenotypes (fragment presence/absence) were fixed or nearly fixed in the two strains (for example, fragment present/absent for all individuals or all individuals except one). Our assumption was that such a pattern of extreme inter-strain genetic differentiation could be explained by the spread, in the resistant strain, of an advantageous allele initially present at low or intermediate frequency in the susceptible strain (standing variation), from which it is eventually purged by genetic drift and/or because it is slightly deleterious.

For 70 such markers, bacterial cultures were sent to Genome Express^® ^http://www.genome-express.com for insert amplification and sequencing with M13 forward and M13 reverse primers. Raw sequence files were edited with BioEdit 7.0.9 [60] and purged from *Pony *and primer sequences. The obtained sequenced were blasted against the full genomic sequence of *Ae. aegypti *(consisting of 4758 supercontigs and available at http://aaegypti.vectorbase.org/GetData/Downloads?type=Genome).

### Identification of candidate genes

Although mechanisms of resistance to *Bti *are still unknown in dipterans, resistances to several Cry toxins have been intensively studied, especially in lepidopteran pests resistant to transgenic crops expressing *Bacillus thuringiensis *Cry toxins genes [20,30]. Because these toxins share similar three-dimensional structures, similar modes of action and resistance mechanisms can be expected between lepidopteran and dipteran insects [16,61]. We therefore considered as candidate genes for *Bti *resistance those belonging to families previously proved to be involved in Cry resistance [20,30,31]. To this list, we added genes potentially implicated in activation of *Bti *toxins (aminopeptidases, e.g. [28]; and trypsins and chymotrypsins, e.g. [62]), in toxin binding (alkaline phosphatases, e.g. [49,63]; aminopeptidases, e.g. [64]; cadherins, e.g. [48]; galactosidases and glycosyltransferases, e.g. [20,65]); or immune defense (mitogen-activated protein kinases, e.g. [31]). A keyword search was conducted in the VectorBase database http://aaegypti.vectorbase.org/index.php and a total of 160 candidates located on 98 different supercontigs were identified out of the 15,419 putative genes (16789 transcripts in total) referenced in the *Aedes aegypti *genome.

### Cadherin (CAD) and leucine aminopeptidase (LAP) gene sequencing

The complete genomic sequences of the CAD and LAP genes (VectorBase Gene IDs AAEL001196 and AAEL001649, respectively) were downloaded from the VectorBase website to help design sequencing primers (Additional file 4) with the software package Lasergene 7.2 (DNASTAR Inc.). The sequencing strategy for the CAD gene targeted exon 5 and more specifically the membrane-proximal subdomains (subdomains 4 to 6) of the protein which are the preferential binding sites of *Bti *Cry toxins in lepidopterans (Figure 1). For the LAP gene, three different primer pairs were selected to amplify the two main exons.

PCR amplifications were conducted for each gene in a 25-μl total volume with 2 mM MgCl2, 0.1 mM of each dNTP (Roche), 0.2 μM of each primer, 5 μg of BSA, 0.6 U of AmpliTaq Gold DNA polymerase (Applied Biosystems) and 10-30 ng of DNA. The PCR program included an initial 10-min denaturation step at 95°C; 40 cycles of denaturation at 95°C for 45s, annealing at the optimal temperature indicated in Additional file 4 for 45s and elongation at 72°C for 60s; followed by a final extension step at 72°C for 5 min. PCR products were purified with the QIAquick PCR purification kit (Qiagen) and sequencing reactions were performed in both directions using the amplification primers and the BigDye Terminator Cycle Sequencing Kit 3.1 (Applied Biosystems), following the manufacturer's indications. Fluorescently labelled sequencing products were run on an ABI PRISM 3100 capillary DNA sequencer (Applied Biosystems) and sequences were analyzed with SeqMan Pro 7.1.0 (DNASTAR Inc.). Overall, we obtained sequences for 11 and 12 individuals of the susceptible and resistant strains, respectively.

### Sequence analysis and neutrality tests

The software DnaSP 5.0 [66] was used to infer haplotype phase and to assess a variety of genetic diversity and differentiation parameters (e.g., nucleotide diversity *p*, haplotype diversity *Hd*, number of segregating sites *S*, *Fst*, etc.) for each gene. Several statistics were also calculated based on the observed polymorphism data to test for deviation from neutral evolution in the resistant strain, including Tajima's *D *[67], Fu and Li's D* and F* [43], and Fay and Wu's *H *[44]. To assess whether these statistics significantly departed from a neutral scenario of evolution given the known demographic history of the resistant strain, we performed coalescent simulations using the program ms [68]. This program generates random independent samples according to a Wright-Fisher neutral model allowing population size changes in the past. For each gene, the mutation rate μ was estimated from the per-locus mutation parameter ? observed for the susceptible strain (θ = 4N_e_μ and N_e _= 6000) and used as the starting value for the simulations (that is, as the value at present). Then 1000 neutral samples consisting of 24 haplotypes were simulated based on the known demographic history of the resistant strain (Additional file 1).

### RNA extraction and gene expression analyses

For the two candidate genes, real-time reverse-transcription PCR (RT-PCR) analyses were performed on three biological replicates for each strain, with each replicate consisting of 30 larvae reared in standard insectary conditions up to the fourth-instar stage (5 days). Total RNAs were extracted using TRIzol (Invitrogen) following the manufacturer's instructions and their quality was assessed with a 2100 Bioanalyzer (Agilent) after DNase I (Invitrogen) treatment. Four micrograms of total RNA were digested with DNase I (Invitrogen) and then used for first-strand cDNA synthesis with SuperScript III (Invitrogen) reverse transcriptase and oligo-dT_20 _primers for 60 min at 50°C, according to the manufacturer's instructions. Real-time RT-PCR reactions were performed on an iQ5 system (Bio-Rad) in a 25-μL total reaction volume with 0.3 μM of each primer, 12.5 μL of iQ SYBR Green supermix (Bio-Rad) and 5 μl of cDNA diluted 25 times. The real-time RT-PCR program included an initial 3-min denaturation step at 95°C and 40 cycles of denaturation at 95°C for 15s and annealing for 30s at the optimal temperature indicated in Additional file 4. For each gene, real-time RT-PCR efficiency was estimated from a serial dilution of cDNA (5 to 500 times) and taken into account in the data analysis performed with the ΔΔC_T _method [69]. Two housekeeping genes encoding ribosomal protein L8 (*RPL8*, GenBank accession number: DQ440262) and S7 (*RPS7*, GenBank accession number: AY380336) were used for normalization. Results were represented as mean expression ratios between *Bti*-resistant and susceptible larvae (± SE).

## Abbreviations

*Bti*: *Bacillus thuringiensis *subsp. *israelensis*; CAD: cadherin; DArT: Diversity Arrays Technology; LAP: leucine aminopeptidase; MITE: miniature inverted-repeat transposable element; RT-PCR: reverse-transcription PCR.

## Authors' contributions

AB carried out the genome scan, analyzed the candidate gene sequence data and drafted the manuscript. MP worked on the outlier sequences, identified the two candidate genes, helped with the analyses and wrote substantial parts of the paper. GT obtained and analyzed the candidate gene sequence and expression data and was involved in the writing. JPD supervised the gene expression study and helped draft the manuscript. LD conceived the overall study, performed the demographic simulations and took part to the data analysis and to the writing. All authors read and approved the final manuscript.

## Supplementary Material

Additional file 1 **Demographic history of the *Aedes aegypti Bti*-resistant strain**. The *Bti*-resistant strain was originally selected from the susceptible standard Bora-Bora strain. This table presents the effective population size at each generation of selection. Click here for file

Additional file 2 **Results of the Dfdist analysis with α = 1%**. In this plot of inter-strain *Fst *values against heterozygosity estimates, each dot represents a DArT marker. The red lines represent the 99% neutral confidence interval simulated using the program Dfdist [26]. Markers situated outside this interval diverge from neutral expectations and are thus potentially under selection. Here, the confidence interval is so large that it includes almost the entire range of possible *Fst *values. Click here for file

Additional file 3 **Outlier markers with a unique localization in the genome of *Aedes aegypti***. This table lists the 41 outlier markers with a unique localization in the genome of *Aedes aegypti*, as well as the numbers of annotated genes and candidate genes in the corresponding supercontigs. Click here for file

Additional file 4 **Primer pairs used for sequencing and real-time RT-PCR analyses**. This table details the different primer pairs used in this study. Click here for file
