# Supplementary material for: Candidate genes revealed by a genome scan for mosquito resistance to a bacterial insecticide: sequence and gene expression variations
Source: BMC Genomics. 2009 Nov 21;10:551. doi: 10.1186/1471-2164-10-551 (PMC2799440; doi:10.1186/1471-2164-10-551)
Supplement: Additional file 1 — Demographic history of the Aedes aegypti Bti-resistant strain. The Bti-resistant strain was originally selected from the susceptible standard Bora-Bora strain. This table presents the effective population size at each generation of selection. [file 1471-2164-10-551-S1.DOC]

## Additional file 1 - Demographic history of the *Aedes aegypti* *Bti*-resistant strain.

The *Bti*-resistant strain was originally selected from the susceptible standard Bora-Bora strain. This table presents the effective population size at each generation of selection.

| **Generation** | **Selection** | **Population effective size** |
| --- | --- | --- |
| 0 (Susceptible strain) | Yes | 6000 |
| 1 | Yes | 300 |
| 2 | Yes | 300 |
| 3 | Yes | 300 |
| 4 | Yes | 300 |
| 5 | Yes | 300 |
| 6 | Yes | 300 |
| 7 | Yes | 300 |
| 8 | Yes | 300 |
| 9 | Yes | 300 |
| 10 | No* | 25 |
| 11 | No* | 1000 |
| 12 | Yes | 1000 |
| 13 | Yes | 1000 |
| 14 | Yes | 1000 |
| 15 | Yes | 1000 |
| 16 | Yes | 1000 |
| 17 | Yes | 1000 |
| 18 | Yes | 1000 |
| 19 | Yes | 1000 |
| 20 (Resistant strain) | - | 2000 |

* The resistant strain experienced a strong decrease in population size after 10 generations of selection, so selection was interrupted for two generations.
