# Supplementary material for: Candidate genes revealed by a genome scan for mosquito resistance to a bacterial insecticide: sequence and gene expression variations
Source: BMC Genomics. 2009 Nov 21;10:551. doi: 10.1186/1471-2164-10-551 (PMC2799440; doi:10.1186/1471-2164-10-551)
Supplement: Additional file 2 — Results of the Dfdist analysis with α = 1%. In this plot of inter-strain Fst values against heterozygosity estimates, each dot represents a DArT marker. The red lines represent the 99% neutral confidence interval simulated using the program Dfdist [26]. Markers situated outside this interval diverge from neutral expectations and are thus potentially under selection. Here, the confidence interval is so large that it includes almost the entire range of possible Fst values. [file 1471-2164-10-551-S2.DOC]

## Additional file 2 – Results of the Dfdist analysis with α=1%.

In this plot of inter-strain *Fst* values against heterozygosity estimates, each dot represents a DArT marker. The red lines represent the 99% neutral confidence interval simulated using the program Dfdist [26]. Markers situated outside this interval diverge from neutral expectations and are thus potentially under selection. Here, the confidence interval is so large that it includes almost the entire range of possible *Fst* values.
