# Supplementary material for: Candidate genes revealed by a genome scan for mosquito resistance to a bacterial insecticide: sequence and gene expression variations
Source: BMC Genomics. 2009 Nov 21;10:551. doi: 10.1186/1471-2164-10-551 (PMC2799440; doi:10.1186/1471-2164-10-551)
Supplement: Additional file 3 — Outlier markers with a unique localization in the genome of Aedes aegypti. This table lists the 41 outlier markers with a unique localization in the genome of Aedes aegypti, as well as the numbers of annotated genes and candidate genes in the corresponding supercontigs. [file 1471-2164-10-551-S3.DOC]

## Additional file 3 - Outlier markers with a unique localization in the genome of *Aedes aegypti*.

This table lists the 41 outlier markers with a unique localization in the genome of *Aedes aegypti*, as well as the numbers of annotated genes and candidate genes in the corresponding supercontigs.

| **Outlier marker** | **Total length (bp)** | **E-Value** | **Length** | **Score** | **Identity (%)** | **Supercontig (chromosome)** | **No. of annoted genes on the supercontig** | **No. of candidate genes on the supercontig** | **Candidate gene (VectorBase Gene ID; distance in bp to the outlier)** |
| --- | --- | --- | --- | --- | --- | --- | --- | --- | --- |
| DArT_223 | 92 | 9E-45 | 92 | 92 | 100.00 | 1.1103 | 1 | 0 |  |
| DArT_237 | 54 | 2E-22 | 54 | 54 | 100.00 | 1.1005 | 1 | 0 |  |
| DArT_279 | 119 | 1E-60 | 119 | 119 | 100.00 | 1.63 | 63 | 0 |  |
| DArT_290 | 126 | 7E-65 | 126 | 126 | 100.00 | 1.113 (2q) | 35 | 0 |  |
| **DArT_318** | **21** | **0.35** | **17** | **17** | **100.00** | **1.38 (3q)** | **43** | **1** | **Leucine aminopeptidase**  **(****AAEL001649; 954948)** |
| DArT_326 | 42 | 2E-15 | 42 | 42 | 100.00 | 1.1644 | 1 | 0 |  |
| DArT_332 | 108 | 3E-54 | 108 | 108 | 100.00 | 1.297 | 15 | 0 |  |
| DArT_371 | 39 | 4E-13 | 38 | 38 | 100.00 | 1.238 | 18 | 0 |  |
| DArT_374 | 77 | 9E-29 | 65 | 65 | 100.00 | 1.474 | 27 | 0 |  |
| DArT_378 | 120 | 2E-25 | 60 | 60 | 100.00 | 1.91 | 19 | 0 |  |
| DArT_385 | 25 | 4E-05 | 24 | 24 | 100.00 | 1.55 | 43 | 0 |  |
| DArT_402 | 45 | 4E-17 | 45 | 45 | 100.00 | 1.505 | 5 | 0 |  |
| DArT_422 | 228 | 1E-125 | 228 | 228 | 100.00 | 1.188 | 18 | 0 |  |
| DArT_425 | 182 | 4E-98 | 182 | 182 | 100.00 | 1.166 | 16 | 0 |  |
| DArT_427 | 28 | 2E-6 | 28 | 28 | 100.00 | 1.62 | 22 | 0 |  |
| DArT_431 | 18 | 0.036 | 18 | 18 | 100.00 | 1.260 | 15 | 0 |  |
| DArT_437 | 84 | 5E-40 | 84 | 84 | 100.00 | 1.868 | 2 | 0 |  |
| DArT_461 | 70 | 9E-32 | 70 | 70 | 100.00 | 1.1562 | 0 | 0 |  |
| **DArT_467** | **255** | **1E-141** | **255** | **255** | **100.00** | **1.25 (2q)** | **30** | **1** | **Cadherin**  **(AAEL001196; 97907)** |
| DArT_469 | 139 | 5E-72 | 138 | 138 | 100.00 | 1.254 | 8 | 0 |  |
| DArT_455 | 245 | 1E-133 | 245 | 241 | 99.59 | 1.176 (1) | 17 | 0 |  |
| DArT_475 | 408 | 1E-102 | 197 | 190 | 99.49 | 1.234 (1) | 11 | 0 |  |
| DArT_445 | 444 | 0 | 349 | 341 | 99.43 | 1.414 | 16 | 0 |  |
| DArT_293 | 281 | 1E-152 | 281 | 273 | 99.29 | 1.43 (2q) | 48 | 0 |  |
| DArT_454 | 460 | 1E-129 | 251 | 235 | 98.41 | 1.195 (2p) | 20 | 0 |  |
| DArT_248 | 464 | 0 | 399 | 369 | 97.49 | 1.172 (2p) | 29 | 0 |  |
| DArT_389 | 198 | 5E-42 | 100 | 88 | 97.00 | 1.40 | 27 | 0 |  |
| DArT_370 | 868 | 0 | 736 | 651 | 96.88 | 1.180 | 29 | 0 |  |
| DArT_242 | 496 | 0 | 416 | 349 | 96.16 | 1.340 | 11 | 0 |  |
| DArT_268 | 167 | 9E-65 | 153 | 126 | 96.1 | 1.376 | 8 | 0 |  |
| DArT_341 | 58 | 9E-16 | 51 | 43 | 96.08 | 1.1416 | 0 | 0 |  |
| **DArT_400** | **76** | **4E-28** | **76** | **64** | **96.05** | **1.85 (3p)** | **43** | **1** | **Mitogen-activated protein kinase (AAEL003359; 297900)** |
| DArT_382 | 109 | 4E-26 | 73 | 61 | 95.89 | 1.365 (3p) | 2 | 0 |  |
| **DArT_432** | **236** | **3E-25** | **72** | **60** | **95.83** | **1.25 (2q)** | **30** | **1** | **Cadherin**  **(AAEL001196; 2213798)** |
| DArT_291 | 174 | 4E-73 | 167 | 140 | 94.83 | 1.369 | 23 | 0 |  |
| DArT_298 | 284 | 5E-34 | 95 | 75 | 94.74 | 1.3092 | 0 | 0 |  |
| **DArT_415** | **108** | **1E-41** | **108** | **87** | **93.52** | **1.1** | **1180** | **1** | **Glycosyltransferase**  **(AAEL000004; 3128011)** |
| DArT_343 | 253 | 2E-94 | 253 | 176 | 91.7 | 1.480 | 12 | 0 |  |
| **DArT_102** | **178** | **2E-56** | **162** | **112** | **90.91** | **1.288 (3p)** | **25** | **1** | **Xaa-pro aminopeptidase**  **(AAEL007892; 907587)** |
| DArT_324 | 221 | 2E-48 | 164 | 99 | 90.36 | 1.151 (2p) | 33 | 0 |  |
| DArT_322 | 112 | 3E-23 | 111 | 56 | 89.19 | 1.724 | 3 | 0 |  |

Markers situated on the same supercontig as a candidate gene are indicated in bold.
