# Supplementary material for: Candidate genes revealed by a genome scan for mosquito resistance to a bacterial insecticide: sequence and gene expression variations
Source: BMC Genomics. 2009 Nov 21;10:551. doi: 10.1186/1471-2164-10-551 (PMC2799440; doi:10.1186/1471-2164-10-551)
Supplement: Additional file 4 — Primer pairs used for sequencing and real-time RT-PCR analyses. This table details the different primer pairs used in this study. [file 1471-2164-10-551-S4.DOC]

## Additional file 4 - Primer pairs used for sequencing and real-time RT-PCR analyses.

This table details the different primer pairs used in this study.

| **Gene** | **Analysis** | | **Primer names** | | **Sequence (5'-3')** | | | | **Tm (°C)** | | **Length (b)** | **Expected product length (bp)** | **Optimal temperature (°C)*** |  |
| --- | --- | --- | --- | --- | --- | --- | --- | --- | --- | --- | --- | --- | --- | --- |
|  |  | |  | |  | | | |  | |  |  |  |  |
| Cadherin (CAD) | Sequencing | | Ae-Cadh-Up6 | | CATTGTGGAAGTTTACGGTGTTTG | | | | 55.2 | | 24 | 728 | 56.6 |  |
|  |  | | Ae-Cadh-Do6 | | GTCGTTGATTCCTTCGTTGTCTGT | | | | 56.1 | | 24 |  |
| Cadherin (CAD) | Sequencing | | Ae-Cadh-Up9 | | AACGGGACTGCCTATTTTTG | | | | 51.1 | | 20 | 586 | 54.6 |  |
|  |  | | Ae-Cadh-Do9 | | TTCGTCGTTTTCTGATGTGTAAG | | | | 51.5 | | 23 |  |
| Cadherin (CAD) | Sequencing | | Ae-Cadh-Up10 | | ATGGGTTTCGCTTTCGGATTC | | | | 56.8 | | 21 | 400 | 56.5 |  |
|  |  | | Ae-Cadh-Do10 | | GTGAGAGTTGCGGTGGCTGTTC | | | | 58.4 | | 22 |  |
| Cadherin (CAD) | Sequencing | | Ae-Cadh-Up11 | | CGACAAGGCCGGAAAGAGCA | | | | 59.8 | | 20 | 673 | 58.4 |  |
|  |  | | Ae-Cadh-Do11 | | CGTGTCCCAATCGTCCAAATCA | | | | 59.2 | | 22 |  |
| Cadherin (CAD) | Real-time RT-PCR | | Ae-Cadh-Up5 | | CGCCAATTCTCACAGTAACC | | | | 54.9 | | 20 | 95 | 49.7 |  |
|  |  | | Ae-Cadh-Do5 | | GAACTTATCCGCTCCATATCC | | | | 54.0 | | 21 |  |
|  |  | |  | |  | | | |  | |  |  |  |  |
| Leucine aminopeptidase (LAP) | Sequencing | | Ae-Amin-Up1 | | ACGCCCCCAACTGTATTTATCA | | | | 55.0 | | 22 | 774 | 58.0 |  |
|  |  | | Ae-Amin-Do1 | | TTCAGGTTCAACTCTTTCGCTACG | | | | 56.7 | | 24 |  |
| Leucine aminopeptidase (LAP) | Sequencing | | Ae-Amin-Up2 | | CGAGGCGAAGAGCTGAAGGAGAA | | | | 60.9 | | 23 | 593 | 59.7 |  |
|  |  | | Ae-Amin-Do2 | | TACGGTACCGGCGACAGCAAATC | | | | 61.7 | | 23 |  |
| Leucine aminopeptidase (LAP) | Sequencing | | Ae-Amin-Up3 | | TCCTGGATATGGCTACCCTAACA | | | | 54.4 | | 23 | 617 | 56.4 |  |
|  |  | | Ae-Amin-Do3 | | CTCACCAAGACCTGCTAACAACC | | | | 54.5 | | 23 |  |
| Leucine aminopeptidase (LAP) | Real-time RT-PCR | | Ae-Amin-Up5 | | TCCTGGATATGGCTACCCTAAC | | | | 57.0 | | 22 | 80 | 52.6 |  |
|  |  | | Ae-Amin-Do5 | | GCCGCTGTTGGTCAAGATAG | | | | 57.0 | | 20 |  |
|  |  | |  | |  | | | |  | |  |  |  |  |
| Ribosomal Protein L8 (RPL8) | Real-time RT-PCR | | Ae60sL8-Up2 | | CTGAAGGGAACCGTCAAGCAA | | | | 58.0 | | 21 | 119 | 56.4 |  |
|  |  | | Ae60sL8-Do2 | | TCGGCGGCAATGAACAACT | | | | 57.3 | | 19 |  |
| Ribosomal Protein S7 (RPS7) | Real-time RT-PCR | | AeRPS7-Up2 | | GTTGGAGATGAACTCGGACCTG | | | | 58.2 | | 22 | 87 | 54.6 |  |
|  |  | | AeRPS7-Do2 | | GCCTTCTTGCTGTTGAACTCG | | | | 57.4 | | 21 |  |
|  | |  | |  | |  |  |  | |  | | | | |

*Temperature used for amplification, determined from the Tm of each primer with the software Beacon Designer 5.10 (Premier Biosoft International).
